# Limitations and Challenges in Modeling Diseases Involving Spinal Motor Neuron Degeneration *in Vitro*

**DOI:** 10.3389/fncel.2018.00061

**Published:** 2018-03-06

**Authors:** Monica Bucchia, Samantha J. Merwin, Diane B. Re, Shingo Kariya

**Affiliations:** ^1^Department of Neurology, Columbia University Medical Center, New York, NY, United States; ^2^Department of Environmental Health Sciences, Columbia University Medical Center, New York, NY, United States

**Keywords:** spinal motor neuron, neuromuscular junction, maturation, adult spinal cord, motor neuron culture, amyotrophic lateral sclerosis, sarcopenia, spinal cord injury

## Abstract

Pathogenic conditions involving degeneration of spinal motor neurons (MNs), such as amyotrophic lateral sclerosis, sarcopenia, and spinal cord injury, mostly occur in individuals whose spinal MNs are fully mature. There is currently no effective treatment to prevent death or promote axonal regeneration of the spinal MNs affected in these patients. To increase our understanding and find a cure for such conditions, easily controllable and monitorable cell culture models allow for a better dissection of certain molecular and cellular events that cannot be teased apart in whole organism models. To date, various types of spinal MN cultures have been described. Yet these models are all based on the use of immature neurons or neurons uncharacterized for their degree of maturity after being isolated and cultured. Additionally, studying only MNs cannot give a comprehensive and complete view of the neurodegenerative processes usually involving other cell types. To date, there is no confirmed *in vitro* model faithfully emulating disease or injury of the mature spinal MNs. In this review, we summarize the different limitations of currently available culture models, and discuss the challenges that have to be overcome for developing more reliable and translational platforms for the *in vitro* study of spinal MN degeneration.

## Limitations

### Immature vs. Mature

Degeneration of matured spinal motor neurons (MNs) and their peripheral axons occurs in neurodegenerative disorders [e.g., amyotrophic lateral sclerosis (ALS)] (Kinsley and Siddique, 1993; Peters et al., 2015), as well as in aging (i.e., sarcopenia) (Aagaard et al., 2010; Berger and Doherty, 2010; Drey et al., 2014), and following spinal cord injury (Koliatsos et al., 1994; Li et al., 1995). There is currently no treatment that can effectively prevent death and promote axonal regeneration of the spinal MNs affected in these patients. To study such conditions, easily controllable and monitorable cell culture models allow for a better dissection of certain aspects of the disease mechanisms (e.g., direct live monitoring of cellular phenotypic changes or molecular cascades in response to stimuli) that are difficult to perform within a whole organism. In the past, many important progresses were made through *in vitro* studies utilizing primary spinal MNs isolated from mouse embryos or MNs differentiated from embryonic stem (ES) cells. However, these neurons are literally in their immature state (Arbab et al., 2014), raising the question of whether they are appropriate for modeling diseases that befall adults whose spinal MNs are fully matured. The groundbreaking discovery of the method for reprogramming differentiated cells into stem cells [induced pluripotent stem (iPS) cells] enabled us, for the first time, to unlimitedly expand and study MNs derived from adult patients (Takahashi and Yamanaka, 2006; Dimos et al., 2008; Li et al., 2008). While they obviously present significant advantages as a personalized and relatively non-invasive source of central nervous system (CNS)-relevant human material, a recent transcriptome study has revealed that iPS cell-derived MNs (iMNs) are closer to embryonic spinal MNs than to their freshly isolated adult counterparts (Ho et al., 2016). This finding brought a lot of disappointment to the field by highlighting that these adult-derived cells also share the same drawbacks as other currently available sources of MNs.

How do mouse spinal MNs differentiate and mature *in vivo*? At embryonic day (E) 9.5, gradient signals of two morphogens (retinoic acid and sonic hedgehog) created in the neural tube organize topographical positioning of neural precursor cells and induce their differentiation into spinal MNs (Pierani et al., 1999; Appel and Eisen, 2003; Novitch et al., 2003). These immature neurons start to express specific transcription factors, such as the homeobox gene *HB9* and the LIM homeodomain genes *Isl1* and *Lhx3*, crucial for the spinal MNs to gain potential for projecting axons to peripheral muscles and releasing neurotransmitters (Wichterle et al., 2002; Rhee et al., 2016). After around E13, axonal terminals contacted to their muscle targets begin to form neuromuscular synapses [neuromuscular junctions (NMJs)], maturation of which is mostly completed by the end of the third postnatal week (Lichtman and Sanes, 2003; Kariya et al., 2008; Shi et al., 2012). During this period (E13-P21), the NMJ post-synapse, which is initially poly-innervated by multiple spinal MNs, becomes mono-innervated by a single spinal MN in the phenomenon known as axonal elimination (Culican et al., 1998; Keller-Peck et al., 2001; Walsh and Lichtman, 2003), and the NMJ pre-synapses that survived this competition gradually mature into a complex elaborated structure (Lichtman and Sanes, 2003; Shi et al., 2012). This process proceeds in morphological alignment with post-synaptic maturation from small and plaque-like to large pretzel shapes (Sanes and Lichtman, 2001; Lichtman and Sanes, 2003; Kariya et al., 2008; Shi et al., 2012). At NMJ pre-synapses, voltage-gated calcium channels are primarily N types (Cav2.2) in a neonatal stage (Rosato Siri and Uchitel, 1999; Santafé et al., 2001; Rosato-Siri et al., 2002; Urbano et al., 2003), but by P14 are eventually replaced and dominated with mature P/Q types (Cav2.1) (Uchitel et al., 1992; Bowersox et al., 1995; Katz et al., 1996; Rosato Siri and Uchitel, 1999; Nudler et al., 2003; Chand et al., 2015). Spinal MNs cannot engage in these maturation processes cell-autonomously, but through temporally regulated bidirectional interactions with muscle cells (Grinnell, 1995; Urbano et al., 2002; Lichtman and Sanes, 2003; Shi et al., 2012). Therefore, if simply plated as a sole pure population, cultured spinal MNs will never gain true maturity, even after long-term maintenance.

### Regenerating vs. Settled

*In vivo*, peripheral axons of fully matured spinal MNs barely lengthen, but terminate at muscle fibers and form NMJs. The axonal terminals of these “settled” spinal MNs display highly specialized, large, and branched structures (NMJ pre-synapses) to efficiently and promptly execute their energy-consuming tasks, such as neurotransmitter release and vesicle recycling (Rash et al., 1988; Betz and Bewick, 1992; Morris and Hollenbeck, 1993; Hoopmann et al., 2012). In contrast, axonal terminals of immature spinal MNs grown *in vitro* manifest a hand-like morphology (growth cones) and are devoted to active regeneration, lengthening in response to neurotrophic factors supplemented in the culture media (Arakawa et al., 1990; Henderson et al., 1994; Estévez et al., 1998). These differences cannot be ignored, especially when the pathomechanisms of conditions attacking the settled spinal MNs *in vivo* are to be modeled and studied *in vitro*. Examples of such conditions include sarcopenia, characterized by an aging-associated pathological loss of muscle mass and strength (Aagaard et al., 2010; Berger and Doherty, 2010; Drey et al., 2014), and the adult-onset fatal neurodegenerative disorder ALS (Kinsley and Siddique, 1993). Mounting evidence indicates that sarcopenia involves the defective maintenance of mature NMJs and subsequent spinal MN death (Chai et al., 2011; Ławniczak and Kmieć, 2012; Tudoraʂcu et al., 2014). While its exact etiology remains to be elucidated, sarcopenia clearly cannot simply be modeled by regenerating immature neurons *in vitro*. This concept would also apply for the *in vitro* modeling of ALS, whose central pathology primarily arises from degeneration of settled MNs (Cappello and Francolini, 2017). To correctly capture disease-relevant pathologies of these conditions in a dish, it may be most optimal from the translational point of view to first create mature NMJs before to start investigations.

The study of human conditions involving spinal MN degeneration can generally be broken down into three significant aspects: (i) the original cause or trigger of the disease (degeneration); (ii) the molecular and cellular cascade responsible for the degenerative process; and (iii) the regenerative mechanisms after the disease onset. An exception may be spinal cord injuries, for which (i) is already known. Studying the process of axonal regeneration in a simple MN mono-culture without muscle cells may sound reasonable, as this is a process prior to spinal MN settlement. However, careful attention still needs to be paid when utilizing embryonic neurons for understanding adult diseases, as their regenerative potential may not be the same as that of adult spinal MNs. The best would be to harvest primary spinal MNs from adult mice. In fact, several protocols detailing the methods for this are available (Milligan and Gifondorwa, 2011; Bektaʂ and Öztürk, 2013; Beaudet et al., 2015); however, they have thus far not being widely replicated. Additionally, the MN yields described in these studies are very low, encompassing the inherent risk of selecting the most resistant spinal MN populations and thus introducing a bias in their use for modeling diseases that in sharp contrast generally affect the most vulnerable populations of MNs.

### Naked vs. Embedded

In the ventral horn, spinal MNs are supported by glial cells at the structural, trophic, and metabolic levels (Blackburn et al., 2009; Christensen et al., 2013). Chondroitin sulfate proteoglycans secreted by astrocytes are the principal constituents of the extracellular matrix, known as perineuronal nets (Murakami and Ohtsuka, 2003; Haggerty et al., 2017), which not only serve as a scaffold for anchoring spinal MNs, but are also actively involved in neural signaling and plasticity (Maeda et al., 2010; Suttkus et al., 2016). Astrocytes also secrete multiple trophic factors regulating spinal MN survival and physiology, such as vascular endothelial growth factor (Van Damme, 2009) and glial cell-derived neurotrophic factor, which is also provided by neurons themselves and peripherally by Schwann cells and muscles via retrograde axonal transport (Springer et al., 1995; Houenou et al., 1996; Zahavi et al., 2015). Peripherally produced insulin-like growth factor-1 crosses the blood–brain barrier and enhances the neuroprotective potential of astrocytes through binding to the receptors expressed on their cell membranes (Ang et al., 1993; Gray et al., 2017). Lactate released by astrocytes is believed to be taken up and utilized as an energy source by neurons (Béland-Millar et al., 2017). Other important functions of astrocytes include clearance of excess glutamate from the inter-synaptic space to prevent neuronal damage due to hyper-excitation (Barres, 1991). Microglia, another type of glial cells in the CNS, serve as the resident innate immune cells to protect CNS tissues from damaging events (e.g., infection, degeneration, and injury) (Loane and Byrnes, 2010; Amor et al., 2014). Finally, oligodendrocytes, the CNS counterpart of Schwann cells, insulate and nourish motor axons via the formation of myelin sheets (Jakovcevski et al., 2009; Saab and Nave, 2017). Spinal MNs “embedded” in the spinal cord *in vivo* receive all these glial benefits in a spatiotemporally regulated manner based on local biological requirements. This situation is quite different from that of dissociated or differentiated MNs harvested “naked” in a plate and forced to regenerate in culture media supplemented with fixed and spatially homogenous concentrations of trophic factors.

Despite the clearly biased environment of cultured spinal MNs *in vitro*, the naked neuron mono-culture is still useful for the investigation of certain biological aspects, such as the capture of disease-related cell-autonomous events. A high-throughput drug screening study that has recently been conducted by utilizing the survival of ALS patient iMNs as readout has identified bosutinib, the Src/c-Abl inhibitor approved by FDA for treating patients with chronic myelogenous leukemia, as a potential therapeutic candidate for ALS (Imamura et al., 2017). Another recent study has found, through deep transcriptome profiling, that maturation and aging-associated pathways are disrupted in sporadic ALS patient iMNs (Ho et al., 2016). In order to understand the pathogenic roles of astrocytes in ALS, we previously cultured control human ES MNs on monolayers of human primary cells obtained from ALS or non-ALS patients, and demonstrated the selective neurotoxic effect of astrocytes, but not fibroblasts, harvested from sporadic ALS patients (Re et al., 2014). Other groups have shown the neurotoxic effect of ALS microglia by using similar *in vitro* co-culture approaches (Liao et al., 2012; Frakes et al., 2014). Thus, co-culture with glial cells, if not a perfect modeling of the *in vivo* condition, can still help in identifying some disease-relevant mechanisms.

## Spinal Motor Neuron Source for Cell Culture

Currently, there are two major spinal MN sources for *in vitro* studies: stem cell-derived MNs and embryonic mouse primary spinal MNs (**Table 1**). The most utilized stem cells for studying MN biology are mouse ES cells and human iPS cells. The main advantages of these cell lines compared to primary neurons are that they can unlimitedly be expanded and stored as homogeneous populations. They are thus suited for studies requiring a large quantity of neurons for repetitive tasks, such as drug screening, proteomics, and biochemistry. On the other hand, primary MNs, which have fully differentiated *in vivo* before being dissociated from the spinal cord, should possess a more genuine identity of spinal MNs compared with MNs differentiated *in vitro* from stem cells. In terms of purity and quantity, however, primary cultures often face variable contamination with other cell types, and the number of viable primary MNs obtainable per embryo is much more limited. Of prime importance, the iPS cells are the only source enabling us to study patient-derived MNs *in vitro* (Chipman et al., 2012). Several groups have succeeded to directly convert human fibroblasts into functional spinal MNs without transiting through a neural progenitor state (Son et al., 2011; Liu et al., 2013); however, improvement in the MN yield has to be achieved before this technique can be routinely utilized.

**Table 1:** General features of currently available spinal motor neurons (MNs) for cell culture.

	Stem cell-derived MN	Dissociated primary MN	Spinal cord explant	Mouse model
MN purity	Good	Not perfect	Presence of other cell types as seen *in vivo*	**N/A**
Application	• Mechanisms in a single MN • Screening	• Mechanisms in a single MN • Screening	• Mechanisms in an integrated system	• Various *in vivo* studies • Cells for *in vitro* studies
Advantage	• Expandable and storable • Sortable • Human iPS cell: human-derived and personalized	• Sortable	• Retains micro-environment of MNs *in vivo* • Requires less supplemental control	• Most translational
Disadvantage	• Could be less physiologic as compared to primary MNs	• Variable contamination of other cell types	• Not sortable • Less quantifiable	• Time consuming • High cost for maintenance
	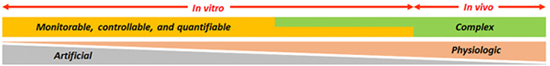			

As an alternative to “naked” neuron cultures, primary spinal MNs can also be harvested as a form of organotypic spinal cord slices (**Table 1**). With this method, freshly dissected thin cross sections of the spinal cord are directly explanted in a dish. Thus, spinal MNs “embedded” in the explant do not undergo harsh procedures of chemical digestion and mechanical dissociation, but regenerate *in vitro* within a micro-environment resembling the *in vivo* condition. This culture system would also allow us to minimize the supplemental control of trophic factors as glial cells integrated in the system may supply them upon local requirements. Noteworthy, the freshly prepared spinal cord culture can serve as a ready-to-use platform for investigating neural networks by electrophysiological recording (Streit and Lüscher, 1992; Ulrich et al., 1994; Tscherter et al., 2001; Magloire and Streit, 2009). One disadvantage of the organotypic slice is that, even with the use of fluorescently labeled MNs, the dense axons freely outgrowing from the spinal MNs packed in the explant often make it difficult to perform a precise single neuron analysis. The motor axons need to be also distinguished from axons from other spinal neurons, such as interneurons (Avossa et al., 2003). Additionally, the intercellular relationships within the explant can eventually be disrupted during a long-term culture, losing the initial *in vivo*-like conditions.

## Challenges

### MN-Muscle Co-culture

Knowing the significant applications that an *in vitro* model of mature NMJs could have, many investigators have attempted in the past to attain this objective (Braun et al., 1996, 1997; Daniels et al., 2000; Mars et al., 2001; Jevsek et al., 2004; Lanuza et al., 2006; Das et al., 2007; Guo et al., 2010, 2011; Lozano et al., 2016; Vilmont et al., 2016). In addition, the recently introduced technologies to differentiate stem cells into MNs have urged researchers to validate the potential of stem cell-derived MNs to form functional NMJs with muscle cells (Boza-Morán et al., 2015; Demestre et al., 2015; Toma et al., 2015; Yoshida et al., 2015; Steinbeck et al., 2016). What has emerged from these co-culture studies is that, regardless of the origin of MNs (i.e., primary or ES/iPS cell-derived), the extent to which NMJs can mature within a certain period of time is much less in a dish than inside the body. One crucial factor restricting the bona fide maturation of NMJs in the conventional MN-muscle cell co-culture is the biological limitation associated with the culture in a single medium or dish of different cell populations with different needs, precluding their healthy growth and maintenance. Considering that spinal MNs *in vivo* extend their axons into an environment different from that of their cell bodies, it is more reflective of the physiologic state to culture axons in a compartment distinct from their somas, but with muscle cells. For this reason, a growing number of studies employ a double-compartment micro-fluidic culture system, in which neurons plated in one compartment extend their axons through microgrooves into another compartment where muscle cells are growing (Campenot, 1977; Park et al., 2013; Southam et al., 2013; Zahavi et al., 2015; Uzel et al., 2016). By allowing manipulation of neurons and muscle cells separately in respective compartments, this system permits maximizing muscle cells to efficiently mature into large myofibers, the condition essential for the MN maturation (Sanes and Lichtman, 2001; Lichtman and Sanes, 2003; Shi et al., 2012). Another factor missing in the current models is the third key player for NMJ maturation: Schwann cells. These peripheral glial cells not only nourish MNs through formation of myelin sheaths along their motor axons, but also specialize into the one called terminal Schwann cell, which locally supports NMJ maturation and maintenance (Sanes and Lichtman, 1999; Feng et al., 2005; Sugiura and Lin, 2011; Darabid et al., 2014; Lee et al., 2017). Despite multiple studies demonstrating the beneficial effect of Schwann cells on neuroregeneration in conventional co-culture systems (Ullian et al., 2004; Li et al., 2007; Gingras et al., 2008; Ragancokova et al., 2009; Gerardo-Nava et al., 2014; Hyung et al., 2015; Suh and Hyung, 2018), no study has thus far succeeded in incorporating Schwann cells into the *in vitro* model of NMJs: this difficulty can presumably be overcome by adopting a compartmented culture method as discussed above. While some progresses have been reported, the precise procedures (the timing for plating individual cell types, the culture medium formulation for each compartment at different time points, etc.) for establishing healthy mature and fully functional NMJs in compartmented systems are still under development.

### Adult Primary Spinal MN Culture

What if we could achieve robust regeneration of adult mouse-derived mature spinal MNs *in vitro*? Difficulty in culturing these “grown-up” neurons is thought to be associated with their phenotypic changes. As animals grow, MN dendrites in the ventral horn become longer and form a more complex structure, which increases the chance of MNs to receive more injuries during the isolation, leading to their excitotoxicity-associated cell death (Lu et al., 2000; Momeni and Jarahzadeh, 2012; Rishal and Fainzilber, 2014). Another possibility is related to a decline in their intrinsic capacity for survival: unlike embryonic MNs, adult MNs are highly reliant on peripherally connected cells, such as Schwann cells and muscles, for their survival, making them terribly prone to death when trophic supports from these cells are suddenly shutdown after dissection (Lowrie and Vrbová, 1992; Oorschot and McLennan, 1998; Beck et al., 2001). As an approach to suppress such early death of MNs harvested *in vitro*, Bektaʂ and Öztürk (2013) have described the merit of pre-incubating plated MNs for a few days in a cold medium prior to induce axonal regeneration, while other study has shown the effectiveness of calcium channel blockers (Momeni and Jarahzadeh, 2012). Applying such findings to the organotypic adult spinal culture prepared in a compartmented system with muscle and Schwann cells in the periphery may give rise to more vivid MN regeneration. Overcoming this challenge would be extremely significant: (1) it will allow us to directly analyze various aspects of already-matured spinal MNs *in vitro*; (2) we will be able to compare and study, for the first time *in vitro*, spinal MNs from different stages of maturity (e.g., neonatal vs. adult vs. aged) or diseases (e.g., pre-symptomatic vs. post-symptomatic); and (3) it will serve as a novel cell source for drug screening. Yet, all these exciting applications are based on an assumption that adult spinal MNs plated *in vitro* continue to retain their “mature” properties previously acquired *in vivo*. Therefore, following successful isolation, it is important to verify whether these adult neurons remain as expected or retrieve “immature” properties during regeneration in an artificial *in vitro* environment.

## Conclusion

Spinal MNs cannot truly mature and age alone. They need good supports and influences from multiple neighbors, or say “coworkers,” like CNS glia and peripheral residents, such as Schwann cells and muscle cells. All these cells constituting the neuromuscular system benefit the MNs either directly or indirectly. Incidents or diseases sabotaging their good relationships may disrupt the healthy natural maturation and/or aging of spinal MNs, and often result in motor disorders of various severities. The different limitations that we have raised and discussed here apply not only to spinal MN cultures but also to *in vitro* models of many other cell types. Each culture model has different advantages and disadvantages; it is not a matter of which one is superior to the other. What is important is to select the one most fitted for individual objectives. The good news is that the range of possible models is widening, thanks to recent advance in technologies. Nonetheless, there is certainly still quite some room for progress, and further challenges have to be addressed toward establishing more reliable and translational *in vitro* platforms.

## Author Contributions

MB, SJM, DBR, and SK contributed to devising, editing, and revising of the manuscript.

## Conflict of Interest Statement

The authors declare that the research was conducted in the absence of any commercial or financial relationships that could be construed as a potential conflict of interest.
